# Correction: Synthesis and evaluation of nuciferine and roemerine enantiomers as 5-HT_2_ and α_1_ receptor antagonists

**DOI:** 10.1039/c8md90012d

**Published:** 2018-03-07

**Authors:** Hui Li Heng, Chin Fei Chee, Sek Peng Chin, Yifan Ouyang, Hao Wang, Michael J. C. Buckle, Deron R. Herr, Ian C. Paterson, Stephen W. Doughty, Noorsaadah Abd. Rahman, Lip Yong Chung

**Affiliations:** a Department of Pharmacy , Faculty of Medicine , University of Malaya , 50603 Kuala Lumpur , Malaysia . Email: buckle@um.edu.my ; Email: chungly@um.edu.my ; Fax: +60 3 79674964 ; Tel: +60 3 79674959; b Department of Chemistry , Faculty of Science , University of Malaya , 50603 Kuala Lumpur , Malaysia; c School of Pharmacy , Ningxia Medical University , Yinchuan , 750004 , P. R. China; d Department of Pharmacology , Yong Loo Lin School of Medicine , National University of Singapore , 117597 , Singapore; e Department of Oral Biology and Craniofacial Sciences and Oral Cancer Research and Coordinating Centre , Faculty of Dentistry , University of Malaya , 50603 Kuala Lumpur , Malaysia; f Penang Medical College , 4 Jalan Sepoy Lines , 10450 George Town , Pulau Pinang , Malaysia

## Abstract

Correction for ‘Synthesis and evaluation of nuciferine and roemerine enantiomers as 5-HT_2_ and α_1_ receptor antagonists’ by Hui Li Heng *et al.*, *Med. Chem. Commun.*, 2018, DOI: 10.1039/c7md00629b.



## 

The authors regret that Scheme 1 showed the wrong structure for **12a**, **12b**, **3a** and **3b**. Please find below the corrected scheme.

**Scheme 1:**
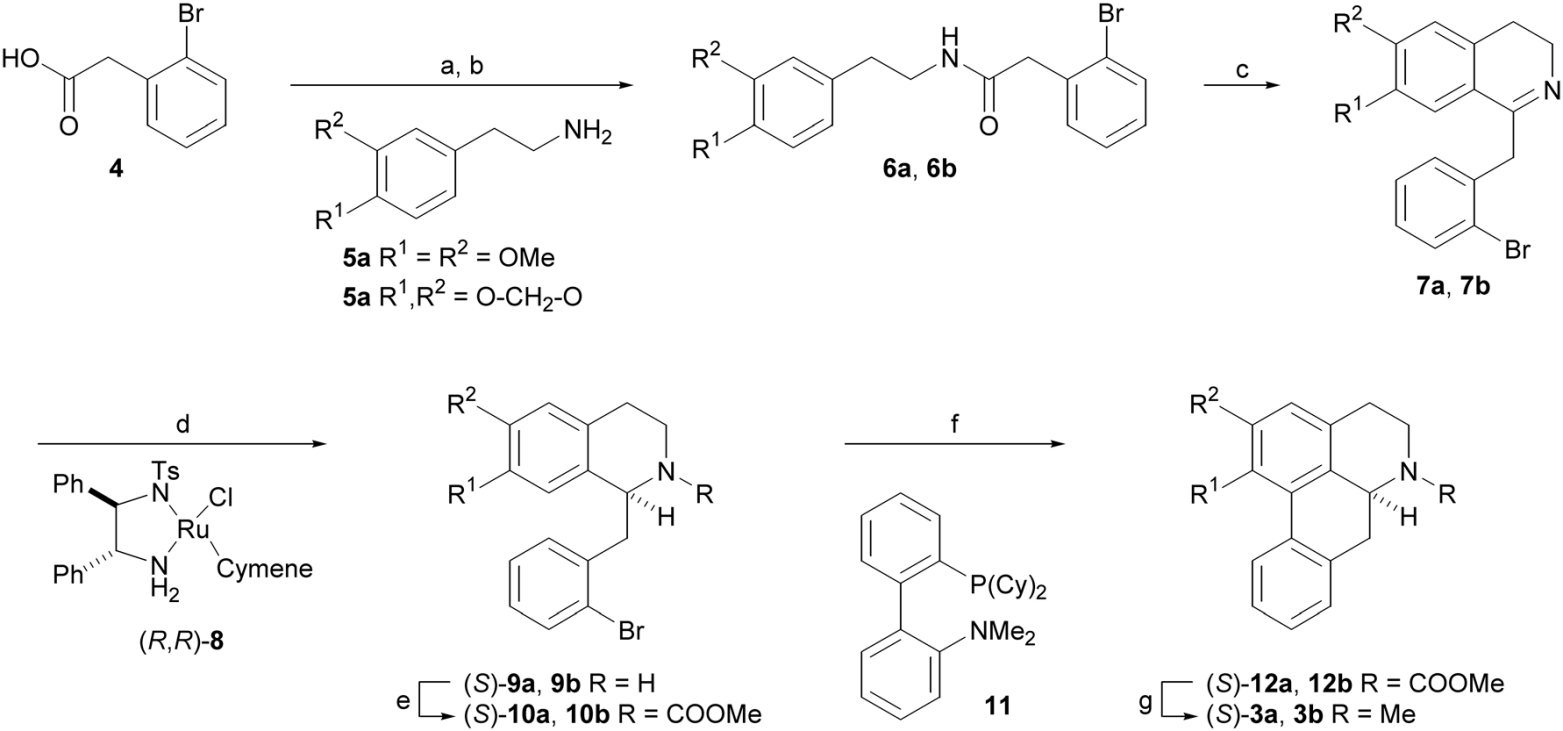


In addition, there were some errors in the numbering of two of the amino acids in Fig. 4, panels C and D. Please find below the corrected figure, including corrected caption.

**Fig. 4:**
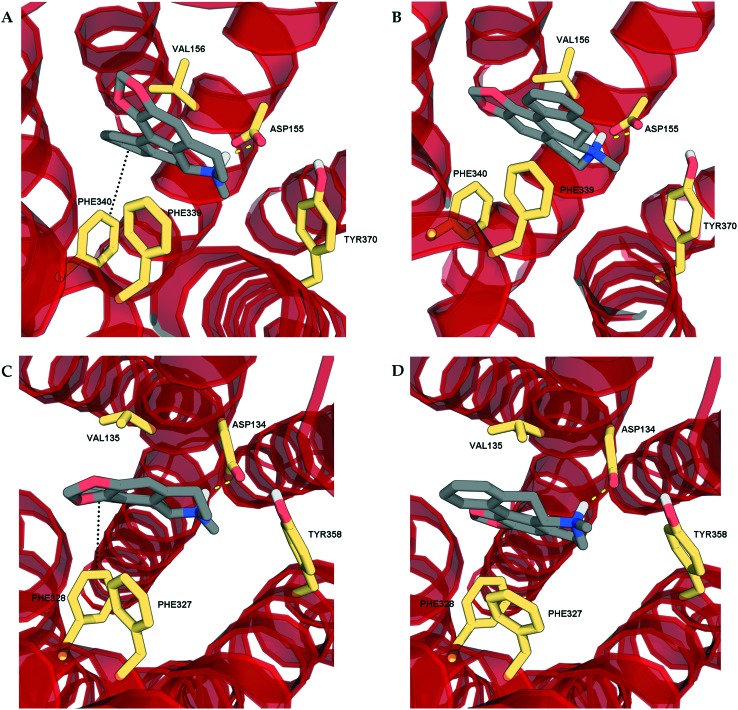
The docking poses of the two enantiomers of roemerine in complex with the 5-HT_2A_ and 5-HT_2C_ receptors. A and C. The poses of (*R*)-roemerine enable a π–π interaction with Phe340/328 as depicted by the black dotted lines. B and D. The poses of (*S*)-roemerine do not allow a π–π interaction with Phe340/328. For the purpose of clarity, only the principal binding residues are depicted and some of the transmembrane helices are not shown.

The Royal Society of Chemistry apologises for these errors and any consequent inconvenience to authors and readers.

